# Effect of maternal vitamin D supplementation on nasal pneumococcal acquisition, carriage dynamics and carriage density in infants in Dhaka, Bangladesh

**DOI:** 10.1186/s12879-022-07032-y

**Published:** 2022-01-13

**Authors:** Mahgol Taghivand, Lisa G. Pell, Mohammed Z. Rahman, Abdullah A. Mahmud, Eric O. Ohuma, Eleanor M. Pullangyeum, Tahmeed Ahmed, Davidson H. Hamer, Stanley H. Zlotkin, Jonathan B. Gubbay, Shaun K. Morris, Daniel E. Roth

**Affiliations:** 1 Department of Nutritional Sciences, University of Toronto, Toronto, Canada; 2 Centre for Global Child Health & Child Health Evaluative Sciences, Hospital for Sick Children, 686 Bay Street, Toronto, ON Canada; 3 Infections Diseases Division & Nutrition and Clinical Services Division, icddr,b, Dhaka, Bangladesh; 4 Centre for Tropical Medicine and Global Health, Nuffield Department of Medicine, University of Oxford, Oxford, UK; 5 Dalla Lana School of Public Health, University of Toronto, Toronto, Canada; 6 Department of Global Health, Boston University School of Public Health, Boston, USA; 7 Section of Infectious Diseases, Department of Medicine, Boston University School of Medicine, Boston, USA; 8 Department of Pediatrics, Hospital for Sick Children & University of Toronto, 686 Bay Street, Toronto, ON Canada; 9 Public Health Ontario, Toronto, ON Canada

**Keywords:** Pneumococcal carriage, Vitamin D, Maternal supplementation, Infants, Bangladesh

## Abstract

**Background:**

Invasive pneumococcal disease is a major cause of infant morbidity and death worldwide. Vitamin D promotes anti-pneumococcal immune responses in vitro, but whether improvements in infant vitamin D status modify risks of nasal pneumococcal acquisition in early life is not known.

**Methods:**

This is a secondary analysis of data collected in a trial cohort in Dhaka, Bangladesh. Acute respiratory infection (ARI) surveillance was conducted from 0 to 6 months of age among 1060 infants of women randomized to one of four pre/post-partum vitamin D dose combinations or placebo. Nasal swab samples were collected based on standardized ARI criteria, and pneumococcal DNA quantified by qPCR. Hazards ratios of pneumococcal acquisition and carriage dynamics were estimated using interval-censored survival and multi-state modelling.

**Results:**

Pneumococcal carriage was detected at least once in 90% of infants by 6 months of age; overall, 69% of swabs were positive (2616/3792). There were no differences between any vitamin D group and placebo in the hazards of pneumococcal acquisition, carriage dynamics, or carriage density (p > 0.05 for all comparisons).

**Conclusion:**

Despite in vitro data suggesting that vitamin D promoted immune responses against pneumococcus, improvements in postnatal vitamin D status did not reduce the rate, alter age of onset, or change dynamics of nasal pneumococcal colonization in early infancy.

*Trial registration* Registered in ClinicalTrials.gov with the registration number of NCT02388516 and first posted on March 17, 2015.

**Supplementary Information:**

The online version contains supplementary material available at 10.1186/s12879-022-07032-y.

## Background

*Streptococcus pneumoniae* (pneumococcus) is one of the leading bacterial causes of meningitis, sepsis, pneumonia and otitis media in young children worldwide, with the burden falling on low- and middle-income countries (LMICs) [1]. Pneumococcus is not a common cause of neonatal infection in high-income countries, but a study of neonatal infections in South Asia identified pneumococcus as a culture-confirmed cause of neonatal sepsis almost as commonly as *Streptococcus agalactiae* (group B streptococcus) in infants 0–59 days of age [2]. A 2011 review reported pneumococcus accounting for 6% of pathogenic bacteria isolated among cases of community-acquired neonatal sepsis in LMICs [3].

One or more serotypes of pneumococcus typically colonize the nasopharynx in childhood [4, 5]. Ongoing colonization (‘carriage’) is the presence of pneumococcus on a mucosal surface without tissue invasion or damage. Onset of colonization by a serotype (‘acquisition’) may occur as early as the immediate postnatal period; however, carriage status in an individual can change over time (‘carriage dynamics’), therefore an individual may be colonized by multiple serotypes concurrently, and some serotypes may persist long-term while others are lost and replaced by different serotypes [5–8]. Carriage is asymptomatic but can be an antecedent to otitis media and invasive pneumococcal disease (IPD), usually attributable to a recently acquired serotype [9]. Factors influencing progression from carriage to disease include the virulence of the colonizing serotype (certain serotypes are more likely to cause IPD), respiratory virus co-infections, and host immune status [6–8].

Introduction of pneumococcal conjugate vaccines (PCV), which protect against the most common IPD-causing serotypes, has substantially reduced the carriage rates of vaccine-specific serotypes and lowered the incidence of vaccine serotype-related otitis media and IPD; however, overall rates of nasopharyngeal colonization have not decreased owing to the replacement of vaccine-specific serotypes with non-vaccine serotypes [10–12]. Disease occurs most commonly due to recently acquired serotypes in the nasopharynx [9]. Thus, carriage is often used as a trial endpoint with acquisition being of particular interest [13]. Since vaccine studies show the prevention of acquisition of vaccine serotypes reduces the risk of IPD, delaying early-life nasopharyngeal acquisition of all serotypes may reduce the overall infant IPD burden. High pneumococcal density (bacterial load of pneumococcus in the nasopharynx) is associated with lower respiratory tract infections (LRTI) and pediatric pneumonia [14]. Mouse models also show increasing pneumococcal density in the nasal cavity facilitates pneumococcal transmission to susceptible mice [15]. Since PCV vaccination does not begin before 6 weeks of age and infants remain unprotected against several vaccine serotypes until after their second dose (typically given at 10 weeks in many LMICs) [16–18], alternative strategies may reduce IPD risk in the first few months of life.

Vitamin D (VD) supplementation is a candidate preventive intervention against IPD based on its observed anti-pneumococcal effects in in vitro studies where VD stimulated maturation of dendritic cells, reduced pro-inflammatory cytokines, and increased pneumococcal killing [19–21]. However, studies examining the effect of prenatal VD supplementation on newborn immune function produced inconsistent results [22–25]. Observational studies in children found VD insufficiency and deficiency associated with longer recovery times from pneumonia, increased pneumonia severity and increased risk of acute respiratory infections (ARI) [26–29]. Yet, results from randomized controlled trials of VD supplementation as a prevention or treatment method for ARI in children are mixed [30–33]. No studies directly addressed whether VD affects pneumococcal carriage in young children. A postnatal delay in nasopharyngeal pneumococcal acquisition due to improved maternal-infant VD status could be beneficial in reducing the risk of early-infant IPD.

Bangladesh is a LMIC with a high burden of IPD [34] and a high prevalence of maternal and neonatal VD deficiency [35]. In this sub-study of a randomized controlled trial and nested cohort study in Bangladesh [33, 35], we aimed to estimate the dose-dependent effect of maternal VD supplementation during pregnancy and lactation on the risk of nasal pneumococcal acquisition, carriage dynamics (cycling between negative and positive carriage) and nasal pneumococcal carriage density (copies/mL) in infants from birth to 6 months of age.

## Methods

### Study design and participants

This was a secondary analysis of data collected during the Maternal Vitamin D Supplementation to Prevent Acute Respiratory Infections (MDARI) study nested in the Maternal Vitamin D for Infant Growth (MDIG) trial (ClinicalTrials.gov:NCT02388516; First Registered: 17/03/2015). The MDIG trial was a randomized, double-blind, placebo-controlled trial of VD supplementation in pregnancy and the first 6 months postpartum conducted in Dhaka, Bangladesh, where generally healthy women at 17–24 weeks of gestation were randomly assigned to receive 1 of 5 weekly VD supplementation doses: 0 IU during pregnancy and postpartum (group A); 4200 IU (B), 16,800 IU (C) or 28,000 IU during pregnancy only (D); and 28,000 IU during pregnancy and up to 26 weeks postpartum (E). Written informed consent was obtained from all women for participation in the MDIG and MDARI studies, including consent for storage and use of biological specimens. Detailed methods and primary results of the MDIG trial and MDARI study were previously described [33, 35, 36]. The MDARI study was approved by research ethics committees at the International Centre for Diarrhoeal Disease Research, Bangladesh (icddr,b) (ERC protocol PR-14079), and as a MDIG sub-study at the Hospital for Sick Children in Toronto (REB 100039072). All methods were carried out in accordance with relevant guidelines and regulations. The MDARI protocol included pneumococcal carriage detection procedures and a pre-specified aim to examine effects of maternal vitamin D supplementation on infant pneumococcal carriage; however, the aims and statistical analysis plan for the sub-study described in this paper were further developed and finalized after trial data collection and laboratory analyses were completed.

### Data collection methods

Infant ARI episodes were monitored by active and passive surveillance from birth to 6 months, as described previously [33, 35, 36]. Infants with signs of ARI or other illness detected during routine home visits were referred to study physicians for further assessment. Parents were counseled to contact study personnel if infants had signs of ARI (e.g., difficulty breathing, cough, nasal congestion, runny nose, hot to the touch) or were taken to hospital. Collection of a nasal swab specimen was routinely performed when ARI criteria were ascertained beyond the 1st week of life (Additional file 1: Table S1), and/or hospitalization with a diagnosis of pneumonia or bronchiolitis occurred (preferentially during hospitalization or otherwise within 7 days of discharge). Among infants with clinical ARI, a nasal swab was collected if another nasal swab had not been collected in the preceding 7 days and one of these criteria were met: there was at least one study visit in the preceding surveillance week where clinical criteria for ARI were not met, ARI features worsened clinically (i.e., upper respiratory tract infection in the preceding week was classified as a LRTI in the current week), or at least four study weeks passed since the last nasal swab collection. Nasal epithelial cells were collected using a sterile nasal flocked swab (Copan Diagnostics, Inc., model number 56780CS01) stored in universal transport medium (Copan Diagnostics, Inc.) and transported to the Virology Lab at icddr,b in an insulated cold bag. Samples were stored at − 80 °C until batched nucleic acid extraction. Nasal swab specimens were collected between December 2014 and August 2016. The ten-valent PCV vaccine (PCV10) was introduced into the routine immunization schedule of Bangladesh in March 2015. Infants were eligible for inclusion in this sub-study if at least 1 nasal swab was collected over the observation period. All swabs collected during the 6-month surveillance period, including those collected during the 1st week of life, were included in this sub-study analysis.

Total nucleic acids were extracted from the nasal swab transport medium using the InviMag Pathogen Kit/KF96 (Invitek Molecular, Berlin, Germany) on the Kingfisher Flex 96 (Thermo Fisher Scientific). Extracted nucleic acid was a template for quantification of pneumococcal copy number by real-time PCR amplification of *lytA*, a single-copy gene specific to *S. pneumoniae* strains. The qPCR assays were carried out using the TaqMan™ Gene Expression Master Mix (Applied Biosystems™, Thermo Fisher Scientific), forward primer ACGCAATCTAGCAGATGAAGCA, reverse primer TCGTGCGTTTTAATTCCAGCT, and probe FAM TGCCGAAAACGCTTGATACAGGGAG-MGB. Each 25 µL reaction contained 10 µL nucleic acid template, 12.5 µL master mix, 0.5 µL of 10 µM stock solutions of each of the primers and probe, and 1 µL water. Real time PCR was conducted using the following thermal conditions in an ABI 7500 (Applied Biosystem, USA): 95 °C for 10 min followed by 40 cycles of 95 °C for 15 s and 60 °C for 1 min. Quality of amplification was confirmed by visual inspection of the Ct curve; if the quality of the amplification was suboptimal, the test was repeated or considered negative. Conversions of Ct values to copies/mL (carriage density) were based on standard curves generated by determining the threshold cycle values for DNA templates containing *lytA*. All standards were performed in triplicate, and a negative (no template) and positive (*lytA* plasmid control) control were included in every run. All amplification curves were manually inspected. A sample was considered positive for pneumococcus if the Ct value was < 35 or was ≤ 38 and showed appropriate amplification upon visual inspection of the Ct curve (i.e., sigmoidal shape).

Serum 25-hydroxyvitamin D (25(OH)D) concentration was measured, as described, in blood samples collected from the mother at delivery and 3-months postpartum, cord blood, and from infants at 3 months [35].

### Statistical analysis

The effect of maternal VD supplementation on the rate of initial nasal pneumococcal acquisition in infants was estimated using interval-censored survival modelling assuming initial pneumococcal acquisition occurred in the interval from birth to a positive swab (if the first swab was positive) or in the interval between a negative swab and the first positive swab. The primary analysis included all infants with at least one swab collected, and otherwise applied the intent-to-treat principle. In primary models, day 0 was the earliest entry point for infants, and all infants were assumed to be carriage-negative on day 0. The parametric model was fit to the data and the best fitting underlying parametric distribution (Gompertz) was chosen based on the Akaike’s Information Criterion, goodness of fit plots and the smooth hazard distribution of the placebo group (expected to provide the baseline hazard distribution). The timing and duration of the interval was based on the time of the infant’s first positive swab and the timing of their negative swabs (Additional file 6: Method S1). Results were expressed as hazard ratios (HR) with 95% confidence intervals (95%CI) and the placebo group was the reference group in all analyses.

Effects of maternal VD supplementation on infant nasal pneumococcal carriage dynamics were estimated using a multi-state model composed of two states: negative (pneumococcus absent) and positive (pneumococcus present). All infants were assumed to be negative for pneumococcal carriage at birth (day 0). Each ‘carriage episode’ represented the time spent in one state from when one swab result was generated until the next swab (or censoring). The multi-state model was used to estimate the probability of an infant being in a positive state at day 189 postnatal age (end of scheduled follow-up period) given negative starting state, total average length of time spent in each state, the expected time until an infant’s first positive episode, the HR of switching states in each VD supplementation group versus placebo and the ratio of the likelihood of transitioning from a positive-to-negative state versus negative-to-positive state. The multi-state model utilized a constant transition rate with a constant baseline hazard accounting for interval censoring.

Positive carriage density data were log-transformed due to the right-skewed distribution. The effect of maternal VD supplementation on log-transformed nasal pneumococcal carriage density (log-copies/mL) was examined using linear regression including only the swabs classified as “positive” for carriage. Generalized estimating equations (GEE) and robust standard errors accounted for multiple swabs from the same infant.

Sensitivity analyses tested the robustness of all primary analyses including variations of model fit and model variation including a sensitivity analysis where all infants were assumed carriage-negative at day 7 since infants were not considered at-risk of ARI during the first postnatal week in the MDARI study [33]. To test the robustness of the parametric interval-censored modelling, a Cox Proportional Hazard Model sensitivity test was completed. Analyses were performed using STATA version 15.1 and the *msm* package in R (version 3.4.2) was used for multi-state modeling.

## Results

**Fig. 1:**
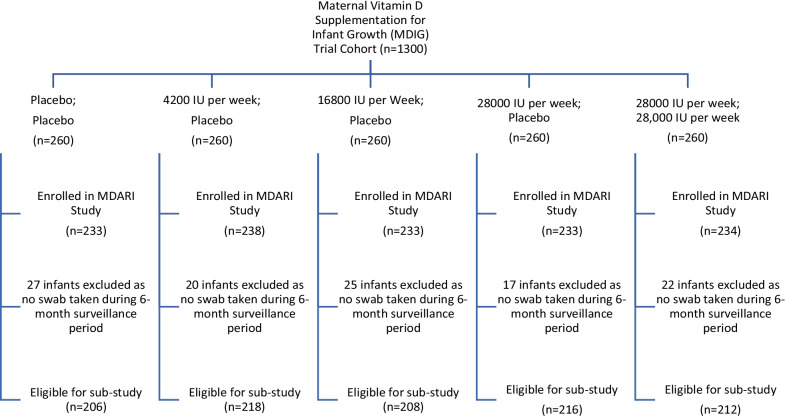
Vitamin D Interventions for Trial Arms and Enrolment of Eligible Infants for Sub-Studies^1^. ^1^Inclusion in current sub-study required the infant to have at least 1 swab taken during the study period. The inclusion/exclusion criteria for the MDIG & MDARI studies have been published elsewhere

Of 1174 infants enrolled in the MDARI study, 1060 had at least one nasal swab and were eligible for inclusion in this sub-study (Fig. 1).

Consistent with findings reported for the MDIG cohort [35], maternal and household characteristics at MDARI study enrolment were similar across the VD or placebo groups. There were VD dose-dependent differences in serum 25(OH)D concentration among mothers at delivery and 3 months postpartum (Table 1) and in cord and infant 3-month samples (Table 1), as reported for the MDIG cohort [35]. Other infant characteristics were similar across groups (Table 1).

**Table 1:** Maternal, infant & household characteristics of participants in the pneumococcal carriage sub-study in Dhaka, Bangladesh (N = 1060)

Characteristics	Prenatal; postpartum vitamin D dose (IU/week)					P^1^
	A: 0; 0	B: 4200; 0	C: 16,800; 0	D: 28,000; 0	E:28,000;28,000	
	N = 206	N = 218	N = 208	N = 216	N = 212	
Mother’s age (years), median ± (25th, 75th percentile)	23 (20, 26)	22.5 (20, 25)	22 (20, 25)	22 (20, 26)	22 (20, 26)	0.7
Month at enrollment (maternal), n (%)						0.9
March–May	78 (38)	72 (33)	81 (39)	76 (35)	71 (33)	
June–August	68 (33)	68 (31)	64 (31)	78 (36)	69 (33)	
September–November	29 (14)	44 (20)	32 (15)	30 (14)	37 (17)	
December–February	31 (15)	34 (16)	31 (15)	32 (15)	35 (17)	
Mother’s education, n (%)						0.7
No education or primary incomplete	52 (25)	53 (25)	54 (26)	58 (27)	57 (27)	
Primary complete	31 (15)	33 (15)	21 (10)	32 (15)	35 (17)	
Secondary incomplete	81 (39)	74 (34)	92 (44)	81 (38)	78 (37)	
Secondary complete	42 (20)	58 (27)	41 (20)	45 (21)	42 (19)	
Asset quintile, n (%)						0.4^A^
1 (lowest)	44 (21)	53 (24)	30 (15)	52 (24)	42 (20)	
2	36 (18)	45 (21)	52 (25)	38 (18)	39 (19)	
3	46 (22)	36 (17)	42 (20)	42 (20)	45 (21)	
4	39 (19)	36 (17)	45 (22)	45 (21)	43 (20)	
5 (highest)	41 (20)	47 (21)	38 (18)	36 (17)	43 (20)	
Maternal height (cm), mean ± SD	151.0 ± 5.4	150.7 ± 5.1	150.5 ± 5.4	149.9 ± 5.3	151.6 ± 5.5	0.03
Maternal serum 25(OH)D concentration at delivery (nmol/L), median (25th, 75th percentile)	21.7 (13.7, 30.2)	67.4 (56.7, 80.6)	101.8 (82.1, 116.6)	109.2 (92.5, 128.2)	111.1 (93.9, 132.8)	0.0^B^
Maternal serum 25(OH)D concentration at 3 months (nmol/L), median (25th, 75th percentile)	24.6 (17.3, 36.9)	30.2 (24.1, 36.9)	50.4 (42.4, 56.7)	58.8 (50.2, 67.6)	98.8 (83.4, 111.3)	0.0^C^
Mode of delivery (n, %)						0.3
Vaginal	102 (49.5)	91 (42)	99 (48)	113 (52)	98 (46)	
C-section	104 (50.5)	127 (58)	109 (54)	103 (48)	114 (54)	
Location of delivery (n, %)^2^						0.5
Facility	175 (85)	192 (88)	177 (85)	179 (83)	175 (83)	
Home	30 (15)	26 (12)	30 (14)	37 (17)	37 (17)	
Number of siblings in household (n, %)						0.4
0	90 (44)	107 (49)	98 (47)	102 (47)	96 (45)	
1	78 (38)	87 (40)	76 (37)	87 (40)	87 (41)	
2 or more	38 (18)	24 (11)	34 (16)	27 (13)	29 (14)	
Month of birth, n (%)						0.08
March–May	26 (13)	31 (14)	30 (14)	21 (10)	38 (18)	
June–August	62 (30)	54 (24)	56 (27)	71 (33)	63 (30)	
September–November	76 (37)	67 (31)	80 (39)	72 (33)	59 (28)	
December–February	42 (20)	66 (31)	42 (20)	52 (24)	52 (24)	
Infant sex (n, %)						0.7
Male	97 (47)	119 (55)	104 (50)	110 (51)	106 (50)	
Female	109 (53)	99 (45)	104 (50)	106 (49)	106 (50)	
PCV10 vaccination by 8 weeks, n (%)						0.6
0 Doses	103 (50)	113 (52)	95 (46)	116 (54)	108 (51)	
1 or more doses	103 (50)	105 (48)	113 (54)	100 (46)	104 (49)	
PCV10 vaccination by 12 weeks, n (%)						0.8
0 Doses	82 (40)	86 (39)	74 (36)	82 (38)	86 (41)	
1 or more doses	124 (60)	132 (61)	134 (64)	134 (62)	126 (58)	
Length-for age-Z-score* at birth, mean (SD)	− 0.80 (1.05)	− 0.91 (1.12)	− 0.89 (1.10)	− 1.03 (1.02)	− 0.88 (0.98)	0.5^D^
Weight-for-age-Z-score* at birth, mean (SD)	− 1.12 (0.81)	− 1.29 (0.89)	− 1.14 (0.88)	− 1.33 (0.84)	− 1.14 (0.88)	0.1^E^
Venous cord 25(OH)D (nmol/L) median ± (25th, 75th percentile)	9.9 (6.7,15.9)	36.2 (31.7,41.7)	59.7 (48.9, 68.6)	70.4 (61.9, 82.9)	71.1 (57.2, 81.9)	0.0^F^
Infant 25(OH)D concentrations at 3 months (nmol/L) median ± (25th, 75th percentile)	24.1 (11.1, 49.7)	28.7 (18.3,48.4)	30.9 (20.1, 50.4)	34.2 (26.9, 48.9)	73.9 (61.9, 85.6)	0.0^G^

*Standardized for gestational age & sex using INTERGROWTH-21st Standards (preterm) or WHO Standards (term)

^A^n_0/0_ = 206, n_4,200/0_ = 217, n_16,800/0_ = 207, n_28,000/0_ = 213, n_28,000/28,000_ = 212 for each treatment group, respectively

^B^n_0/0_ = 114, n_4,200/0_ = 117, n_16,800/0_ = 127, n_28,000/0_ = 108, n_28,000/28,000_ = 122 for each treatment group, respectively

^C^n_0/0_ = 101, n_4,200/0_ = 107, n_16,800/0_ = 115, n_28,000/0_ = 100, n_28,000/28,000_ = 109 for each treatment group, respectively

^D^n_0/0_ = 141, n_4,200/0_ = 150, n_16,800/0_ = 144, n_28,000/0_ = 142, n_28,000/28,000_ = 147 for each treatment group, respectively

^E^n_0/0_ = 143, n_4,200/0_ = 151, n_16,800/0_ = 145, n_28,000/0_ = 143, n_28,000/28,000_ = 150 for each treatment group, respectively

^F^n_0/0_ = 85, n_4,200/0_ = 97, n_16,800/0_ = 101, n_28,000/0_ = 89, n_28,000/28,000_ = 90 for each treatment group, respectively

^G^n_0/0_ = 67, n_4,200/0_ = 61, n_16,800/0_ = 58, n_28,000/0_ = 62, n_28,000/28,000_ = 74 for each treatment group, respectively

^1^Parametric and non-parametric tests (ANOVA, Kruska-Wallis & Chi-square) were selected based on data distribution and used to determine p-values

^2^Other/unknown location of delivery was 1 (0.5%) for group A and 1 (0.5%) for group C

**Fig. 2:**
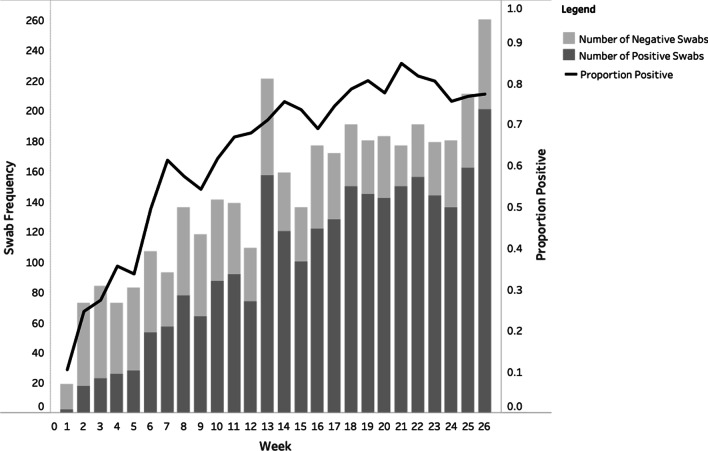
Weekly number and proportion of nasal swabs positive for pneumococcal carriage in infants up to 6 months of age in the MDARI cohort, including all maternal vitamin D supplementation groups (N = 1060)

Of 3792 nasal swab specimens collected, 69% (2616) tested positive for pneumococcus. Overall, 90% (949/1060) of infants were carriage-positive at least once during the first 6 months of life and the number of positive swabs increased with age (Fig. 2).

The total numbers of swabs and proportions positive were similar across VD supplementation groups: 68% (500/737) in Group A, 66% (502/761) in Group B, 70% (507/798) in Group C, 71% (555/781) in Group D and 70% (499/715) in Group E. There was no significant effect of any dose of maternal VD supplementation during pregnancy and postpartum on the relative hazard of pneumococcal acquisition compared to placebo. The median age at first detection of pneumococcal carriage and was similar across intervention groups, ranging from 6.3 to 8.1 weeks (Table 2).

**Table 2:** Effect of maternal vitamin D supplementation on relative hazard of pneumococcal acquisition and pneumococcal density

	Vitamin D supplementation group: prenatal; postpartum dose (IU/week)				
	A: 0;0	B: 4200;0	C: 16,800;0	D: 28,000;0	E: 28,000;28,000
Number of infants with ≥ 1 nasal swab^A^	206	218	208	216	212
Infants ever positive for detection of nasal pneumococcal carriage (≥ 1 positive swab) (%)	186 (90%)	188 (86%)	191 (92%)	193 (89%)	190 (90%)
Relative hazard of pneumococcal acquisition (95% CI)^B^	REF^E^	0.87 (0.70, 1.08)	1.16 (0.94, 1.44)	1.05 (0.85, 1.30)	1.05 (0.84, 1.30)
Median time until 1st detection of pneumococcal carriage (weeks)^B^	7.1	8.1	6.2	6.8	6.8
Positive swabs in pneumococcal density analysis (N)^C^	500	501	560	555	499
Pneumococcal density: percent difference (95% CI)^D^	REF^E^	− 9.7 (− 39, 33)	− 10.7 (− 40, 34)	− 3.4 (− 34, 42)	− 8.9 (− 39, 36)

^A^N = 1060

^B^Estimated using interval-censored parametric modelling assuming a Gompertz distribution

^C^Overall number of positive swabs in each group are the same values

^D^Estimated using log-transformed density data using GEE and robust standard errors to accommodate repeated measurements from the same infants. Analysis based on only samples with detectable pneumococcus

^E^REF indicates that group A (0;0) was the reference group for other estimates

There was no significant effect of maternal VD supplementation during pregnancy and postpartum on infant pneumococcal density for any VD supplementation group compared to placebo (Table 2).

Metrics of nasal pneumococcal carriage dynamics based on multi-state modeling were similar across the VD and placebo groups (Table 3). Specifically, there was no significant effect of VD supplementation on the relative hazard of transitioning from a negative to positive carriage episode or from a positive to negative carriage episode (Table 3). The overall time spent positive across all carriage positive events was similar across the groups, ranging from between 115 to 125 days (Table 3). Among all intervention groups, the probability of pneumococcal carriage was high such that by 120 days of age, there was a 75% probability of a swab being positive (Additional file 7: Fig. S1). The ratio of the risk of progression to positive episode versus recovery from positive episode showed an increasing trend as the VD supplementation dosage increased but confidence intervals overlapped (Table 3).

**Table 3:** Effects of maternal vitamin D supplementation of varying doses on infant nasal pneumococcal carriage dynamics

	Group A	Group B	Group C	Group D	Group E	Overall
Supplementation (prenatal/postpartum)	0/0	4200; 0	16,800; 0	28,000; 0	28,000; 28,000	–
N (infants/swabs)	206/737	218/761	208/798	216/781	212/715	–
Probability of positive status at day 189, % (95% CI)^A,C^	74 (69, 78)	72 (67, 77)	76 (72, 80)	76 (72, 80)	77 (73, 82)	75 (73, 77)
Total time spent negative or positive during all carriage episodes, days (95% CI)^A,C^						
Negative	72.0 (64.4, 80.1)	74.7 (67.5, 82.0)	64.3 (57.7, 71.9)	66.8 (59.4, 74.8)	68.5 (61.8, 76.4)	69.0 (65.7, 72.3)
Positive	117.0 (108.9, 124.6)	114.3 (106.8, 121.6)	124.7 (117.0, 131.3)	122.2 (114.2, 129.6)	120.5 (112.6, 127.2)	120.0 (116.7, 123.3)
Hazard ratio of transitioning between episodes (95% CI)^A,B^						
Negative to positive	REF	0.99 (0.74, 1.33)	1.29 (0.96, 1.74)	1.08 (0.81, 1.46)	0.94 (0.71, 1.26)	–
Positive to negative	REF	1.09 (0.71, 1.67)	1.16 (0.76, 1.78)	0.94 (0.61, 1.46)	0.76 (0.49, 1.19)	–
Expected time until first positive episode, days (95% CI)^A,C^	41.3 (32.7, 51.0)	41.8 (34.2, 52.1)	32.1 (25.8, 40.7)	38.1 (29.9, 47.7)	43.8 (35.2, 53.3)	39.0 (35.5, 43.0)
Ratio of negative to positive episodes: positive to negative episodes^A,C^	2.81 (2.25, 3.71)	2.56 (2.05, 3.14)	3.11 (2.55, 3.91)	3.24 (2.59, 4.21)	3.48 (2.64, 4.07)	3.02 (2.70, 3.34)

^A^Estimated using interval-censored multi-state modelling

^B^REF indicates that group A (0;0) was the reference group for other estimates

^C^Episode is defined as the status of pneumococcal carriage based on the nasal swab until a new nasal swab is taken and a new status is determined

All sensitivity analyses supported the findings of the primary analyses in demonstrating a lack of any significant effects of maternal VD supplementation on pneumococcal carriage acquisition, dynamics or density (Additional file 2: Table S2; Additional file 3: Table S3; Additional file 4: Table S4; Additional file 5: Table S5).

## Discussion

In this sub-study of a randomized placebo-controlled dose-ranging trial in Bangladesh, maternal VD supplementation did not affect the risk of nasal pneumococcal acquisition, dynamics of carriage or carriage density in the infant offspring up to 6 months of age. As IPD is believed to be caused by recently acquired serotypes in the nasopharynx [8], we reasoned if VD supplementation resulted in a delay in pneumococcal carriage acquisition then supplementation could reduce the risk of IPD during the early postnatal period when host immune function is relatively immature and before infants receive at least two PCV doses. The findings indicate improvements in VD status in early infancy do not delay the age of initial postnatal pneumococcal acquisition. To date, few studies have examined the effect of maternal VD supplementation on infant infectious diseases [33], and no trials specifically examined the effects on pneumococcal carriage or IPD. In the context of accumulated evidence, these findings do not support a role for maternal VD supplementation to reduce the risk of IPD in early infancy through reducing the risk of acquisition or effects on carriage dynamics or density.

Maternal VD supplementation in the MDIG trial improved both maternal and infant 25(OH)D levels at birth with sustained effects in the group of infants born to mothers who received 28,000 IU during the postpartum period [35], such that few infants in the highest-dose group had biochemical VD deficiency up to 6 months of age. However, pneumococcal acquisition rates and carriage dynamics were similar in the high-dose prenatal/postpartum group E compared to placebo, and there was no evidence of a beneficial dose–response effect. Several metrics of carriage dynamics were considered in multi-state modeling, and one difference was the ‘ratio of negative-to-positive: positive-to-negative episodes’ was somewhat higher in group E than the other groups (although with overlapping confidence intervals), findings that would suggest a greater rather than reduced tendency to have a positive carriage state in the high-dose VD group. Pneumococcal density was similar in the high-dose prenatal/postpartum group E compared to placebo, and there was no evidence of a beneficial dose–response effect on density.

The anti-pneumococcal immune response promoted by VD in vitro was characterized by enhanced dendritic cell maturation, reduction of pro-inflammatory cytokines and increased neutrophil-mediated killing [19–21]. However, it is possible that these immunomodulating effects of VD are most relevant in the context of a more potent immune stimulus than nasopharyngeal carriage, particularly since innate immune mechanisms may be less important in clearing nasal carriage than in the response to invasive infection [37, 38]. Also, neonatal/infant mouse model studies showed that the clearance of pneumococcal carriage is impaired in neonatal/infant versus adult mice due to reduced macrophage infiltration [39] and lower production of macrophage cytokines involved in neutrophil recruitment and activation [40], suggesting that VD may be more effective in preventing carriage or promoting clearance in older children and adults than in the infants in the present study. However, the deficit in clearing pneumococcal carriage observed in infant mice was ameliorated by immunization [40], and it remains plausible that VD could modify the response to PCV in infants given the known effects of VD on the adaptive immune response [41].

Nearly all infants in the study cohort had pneumococcal acquisition by 6 months of age. Multi-state modeling suggested infants were likely to spend more time in a positive carriage state rather than a negative state, possibly indicating a slower process of clearance of pneumococcus in the nasopharynx compared to the time required to initially acquire pneumococcus or re-acquire it after prior clearance. Cycling between episodes was common throughout the first 6 months of life. In theory, cycling is undesirable if the re-acquisition of pneumococcus results in an increased risk of IPD [9]. However, due to the absence of serotype data in the present study, it was unknown when the infants acquired new serotypes or were recolonized with a serotype acquired in an earlier episode. With increasing age, the probability of being positive for pneumococcal carriage became uncoupled from prior swab status. This observation reinforced the idea that interventions aiming to reduce pneumococcal acquisition in early infancy may not have long-term impacts due to the high overall prevalence of carriage and frequent cycling between positive and negative states. Delaying the age of initial acquisition may not necessarily confer health benefits; acquisition of low-virulence serotypes in early life may reduce the risk of acquisition of pathogenic serotypes due to serotype competition in the nasopharynx through the stimulation of non-serotype specific adaptive immune responses or by limiting the extent to which pathogenic serotypes find an ecological niche for colonization [42–44].

The cohort of infants studied had similar carriage rates at 1, 2 and 3 months of age compared to previous studies conducted in Bangladesh [45–47]. Pneumococcal carriage dynamics were similar to those observed among infants in Thailand [48], but different from non-serotype specific findings in South Africa and Kenya. In the South Africa cohort, the time to acquisition was longer than the current study, but like the current study, 96% of infants were colonized at least once by 260 days of life [49]. In the Kenyan cohort, the carriage duration was shorter than the current study, but 66% of the children were pneumococcal carriers at least once during the longitudinal study [50].

The study was strengthened by leveraging data from a randomized trial where there was a potent effect of supplementation on VD status in a setting of a high prevalence of maternal-neonatal VD deficiency and where nasal pneumococcal acquisition occurs almost universally in early infancy [45–47]. A study limitation was that because swab collection was prompted by ARI criteria, rather than scheduled at specific timepoints, the outcome was conditional on a post-randomization variable. However, we previously showed clinical and virus-associated ARI incidence was unaffected by the VD intervention [33], such that swab collection frequencies were similar across the groups, making bias highly unlikely. Nonetheless, the analyses were based on carriage dynamics during ARI episodes, and it remains possible that our findings would have been different if we had studied carriage during periods in which infants were asymptomatic. As it seemed unlikely that VD supplementation would have a serotype-specific immunomodulatory effect, the resources needed to perform pneumococcal serotyping for the large number of samples were not warranted to address the primary study aims. However, it is plausible that VD could differentially affect serotype acquisition based on its virulence and its propensity to induce an immune response. Serotype data would have enabled a closer examination of carriage dynamics to determine the extent to which negative-to-positive transitions involved newly acquired serotypes.

## Conclusion

Maternal VD supplementation did not delay or reduce the rate of pneumococcal carriage or carriage density in early infancy. However, there may be other mechanisms by which VD supplementation enhances the host response to PCV or reduces the risk or severity of IPD. Therefore, given the anti-pneumococcal effects of VD in vitro, further trials designed specifically to examine the effects of VD supplementation on IPD or to enhance the response to vaccination may be warranted in high-risk populations.

## Supplementary Information

**Additional file 1: Table S1.** MDARI definitions for acute respiratory infections.

**Additional file 2: Table S2.** Effect of maternal vitamin D supplementation on risk of pneumococcal acquisition in infants in Bangladesh using an interval-censored model with a day 7 start.

**Additional file 3: Table S3.** Effects of maternal vitamin D supplementation of varying doses on the risk of pneumococcal acquisition using a Cox proportional hazard model with day 0 start.

**Additional file 4: Table S4.** Effects of maternal vitamin D supplementation of varying doses on the risk of pneumococcal acquisition using a Cox proportional hazard model with day 7 start.

**Additional file 5: Table S5.** Effects of maternal vitamin D supplementation of varying doses on infant nasal pneumococcal carriage dynamics using a multi-state model with day 7 start.

**Additional file 6: Method S1.** Determination of intervals used in interval-censored model.

**Additional file 7: Figure S1.** Probability of change to positive state from a negative state start aggregated across all treatment groups over trial period based on predicted multi-state model probabilities from day 0 to day 189 (N = 3792 swabs).

## Data Availability

De-identified individual participant data that underlie the results reported in this article (text, tables, figures, and appendices) and the study protocol will be available immediately after publication and ending 5 years following article publication. These data and documents will be made available to investigators whose secondary data analysis study protocol has been approved by an independent research ethics board. Proposals should be directed to daniel.roth@sickkids.ca; to gain access, data requestors will need to sign a data access agreement.

## Abbreviations

LMICs: Low- and middle-income countries (LMICs)
IPD: Invasive pneumococcal disease
PCV: Pneumococcal conjugate vaccine
PCV10: Ten-valent pneumococcal conjugate vaccine
LRTI: Lower respiratory tract infections
VD: Vitamin D
ARI: Acute respiratory infections
MDARI: Maternal Vitamin D Supplementation to Prevent Acute Respiratory Infections
MDIG: Maternal Vitamin D for Infant Growth Trial
iccdr,b: International Centre for Diarrhoeal Disease Research, Bangladesh
25(OH)D: Serum 25-hydroxyvitamin D
GEE: Generalized estimating equations
