# Supplementary material for: Effect of maternal vitamin D supplementation on nasal pneumococcal acquisition, carriage dynamics and carriage density in infants in Dhaka, Bangladesh
Source: BMC Infect Dis. 2022 Jan 13;22:52. doi: 10.1186/s12879-022-07032-y (PMC8759256; doi:10.1186/s12879-022-07032-y)
Supplement: Supplementary file 1 — Additional file 1: Table S1. MDARI definitions for acute respiratory infections. [file 12879_2022_7032_MOESM1_ESM.docx]

**Table S1.** MDARI Definitions for Acute Respiratory Infections

| **Acute Respiratory Infection Type** | **Any upper respiratory tract infection or lower respiratory tract infection as defined below** |
| --- | --- |
| Clinical Upper Respiratory Tract Infection (URTI) | A new-onset illness consisting of at least two of the following clinical criteria at any time during a surveillance week:  • Caregiver-reported cough;  • Caregiver-reported rhinorrhea;  • Caregiver-reported nasal congestion;  • Measured temperature ≥37.5 °C (axillary)  confirmed with 2nd measurement |
| Clinical Lower Respiratory Tract Infection (LRTI) | • Caregiver-reported cough AND/OR difficulty breathing during a surveillance week  AND  • Observed elevated respiratory rate (60 breaths per minute or greater for infant up to 59 days of age, or 50 breaths per minute or greater for infant 60 days of age or older) and/or lower chest wall in-drawing  OR  • Hospitalization with physician diagnosis of pneumonia or bronchiolitis |
| Microbiologically Confirmed URTI | URTI that tests positive for at least one of influenza A, influenza B, respiratory syncytial virus, parainfluenza 1, 2, and 3, adenovirus, or human metapneumovirus |
| Microbiologically Confirmed LRTI | LRTI that tests positive for at least one of influenza A, influenza B, respiratory syncytial virus, parainfluenza 1, 2, and 3, adenovirus, or human metapneumovirus |
