# Supplementary material for: Effect of maternal vitamin D supplementation on nasal pneumococcal acquisition, carriage dynamics and carriage density in infants in Dhaka, Bangladesh
Source: BMC Infect Dis. 2022 Jan 13;22:52. doi: 10.1186/s12879-022-07032-y (PMC8759256; doi:10.1186/s12879-022-07032-y)
Supplement: Supplementary file 2 — Additional file 2: Table S2. Effect of maternal vitamin D supplementation on risk of pneumococcal acquisition in infants in Bangladesh using an interval-censored model with a day 7 start. [file 12879_2022_7032_MOESM2_ESM.docx]

**Table S2.** Effect of Maternal Vitamin D Supplementation on Risk of Pneumococcal Acquisition in Infants in Bangladesh

|  | **Vitamin D supplementation group: Prenatal; Postpartum dose (IU/week)** | | | | |
| --- | --- | --- | --- | --- | --- |
|  | **A:** 0;0 | **B:** 4200;0 | **C:** 16800;0 | **D:** 28000;0 | **E:** 28000;28000 |
| **Number of Infants with ≥1 nasal swab** | 206 | 218 | 208 | 216 | 212 |
| **Infants ever positive for detection of nasal pneumococcal carriage**  **(≥1 positive swab) (%)** | 186 (90%) | 188 (86%) | 191 (92%) | 193 (89%) | 190 (90%) |
| **Relative Hazard of Pneumococcal Acquisition (95% CI)^A^** | REF | 0.86 (0.70, 1.07) | 1.16 (0.93, 1.43) | 1.04 (0.84, 1.29) | 1.04 (0.84 1.29) |
| **Median Time Until 1^st^ Detection of Pneumococcal Carriage (Weeks)^A^** | 8.0 | 9.2 | 7.1 | 7.7 | 7.8 |

^A^ Estimated using interval-censored parametric modelling assuming a Gompertz distribution with a day 7 start for infants enrolled prior to 7 days
