# Supplementary material for: Effect of maternal vitamin D supplementation on nasal pneumococcal acquisition, carriage dynamics and carriage density in infants in Dhaka, Bangladesh
Source: BMC Infect Dis. 2022 Jan 13;22:52. doi: 10.1186/s12879-022-07032-y (PMC8759256; doi:10.1186/s12879-022-07032-y)
Supplement: Supplementary file 5 — Additional file 5: Table S5. Effects of maternal vitamin D supplementation of varying doses on infant nasal pneumococcal carriage dynamics using a multi-state model with day 7 start. [file 12879_2022_7032_MOESM5_ESM.docx]

**Table S5.** Effects of Maternal Vitamin D Supplementation of Varying Doses on Infant Nasal Pneumococcal Carriage Dynamics

|  | **Group A** | **Group B** | **Group C** | **Group D** | **Group E** | **Overall** |
| --- | --- | --- | --- | --- | --- | --- |
| **Supplementation**  **(Prenatal/Postpartum)** | 0/0 | 4,200/0 | 16,800/0 | 28,000/0 | 28,000/28,000 | - |
| **N**  **(Infants)** | 206 | 218 | 208 | 216 | 212 | - |
| **Probability of Positive Status at Day 189, % (95% C.I.)^A,C^** | 74  (70, 79) | 73  (68, 77) | 76  (72, 80) | 77  (73, 81) | 78  (73, 82) | 76  (74, 77) |
| **Total Time Spent Negative or Positive during all Carriage Episodes, Days (95% CI)^A,C^** |  |  |  |  |  |  |
| Negative | 69.2  (62.2, 77.2) | 69.2  (62.1, 76.9) | 61.8  (55.0, 69.8) | 63.9  (57.3, 71.1) | 65.5  (58.4, 73.0) | 66.2  (63.1, 69.5) |
| Positive | 119.8  (111.8,126.8) | 119.8  (112.1, 126.9) | 127.2  (119.2, 133.9) | 125.1  (117.9, 131.7) | 123.5  (115.9 130.6) | 122.8  (119.5, 125.9) |
| **Hazard Ratio of Transitioning between Episodes (95% C.I.)^A,B^** |  |  |  |  |  |  |
| Negative to Positive | REF | 1.00  (0.74, 1.35) | 1.30  (0.96, 1.75) | 1.09  (0.81, 1.47) | 0.95  (0.71, 1.27) | - |
| Positive to Negative | REF | 1.11  (0.72, 1.71) | 1.18  (0.77, 1.82) | 0.95  (0.61, 1.48) | 0.77  (0.49, 1.21) | - |
| **Expected Time Until First Positive Episode, Days (95% C.I.)^A,C^** | 38.0  (30.8,47.1) | 37.9  (30.4, 46.4) | 29.3  (23.6, 36.2) | 34.8  (27.9, 43.5) | 40.1  (33.2, 48.2) | 35.7  (32.4, 39.5) |
| **Ratio of negative to positive episodes: positive to negative episodes^A^** | 2.93  (2.21, 3.64) | 2.66  (2.12, 3.33) | 3.23  (2.60, 4.02) | 3.37  (2.66, 4.29) | 3.61  (2.74, 4.71) | 3.12  (2.81, 3.47) |

^A^ Estimated using multi-state modelling with all infants starting on day 7

^B^ Reference group is Placebo

^C^ Episode is defined as the status of pneumococcal carriage based on the nasal swab until a new nasal swab is taken and a new status is determined
