# Supplementary material for: Effect of maternal vitamin D supplementation on nasal pneumococcal acquisition, carriage dynamics and carriage density in infants in Dhaka, Bangladesh
Source: BMC Infect Dis. 2022 Jan 13;22:52. doi: 10.1186/s12879-022-07032-y (PMC8759256; doi:10.1186/s12879-022-07032-y)
Supplement: Supplementary file 6 — Additional file 6: Method S1. Determination of intervals used in interval-censored model. [file 12879_2022_7032_MOESM6_ESM.docx]

**Method S1. Determination of Intervals Used in Interval-censored Model**

The timing and duration of the interval preceding each infant’s first positive swab was based on the postnatal age scale and defined as follows: if an infant did not have any negative swabs before their first positive swab, they were assumed to be negative for pneumococcal carriage at birth and the interval was set as (0, X) with X being the age (in days) of the first positive swab and 0 indicating the assumption of negativity from birth; or, if an infant never had a positive swabs, yet had one or more negative swabs throughout the duration of the follow-up period, they were right censored (Y,.) with Y representing the last known time an infant was negative and the missingness to indicate the infant’s status was unknown at the end of the trial period; or, if an infant had one or more negative swabs prior to their first positive swab, the date of the last negative swab was taken to complete the left side of the interval and the age of the first positive swab was taken to be the right side of the interval (Y, X) indicating the infant first acquired pneumococcal carriage sometime between these two timepoints.
