# Supplementary material for: Effect of maternal vitamin D supplementation on nasal pneumococcal acquisition, carriage dynamics and carriage density in infants in Dhaka, Bangladesh
Source: BMC Infect Dis. 2022 Jan 13;22:52. doi: 10.1186/s12879-022-07032-y (PMC8759256; doi:10.1186/s12879-022-07032-y)
Supplement: Supplementary file 7 — Additional file 7: Figure S1. Probability of change to positive state from a negative state start aggregated across all treatment groups over trial period based on predicted multi-state model probabilities from day 0 to day 189 (N = 3792 swabs). [file 12879_2022_7032_MOESM7_ESM.docx]

**

**Figure S1.** Probability of change to positive state from a negative state start aggregated across all treatment groups over trial period based on predicted multi-state model probabilities from day 0 to day 189 (N=3,792 swabs)
